# Reference values for isometric muscle force among workers for the Netherlands: a comparison of reference values

**DOI:** 10.1186/2052-1847-6-10

**Published:** 2014-02-25

**Authors:** Rob KW Douma, Remko Soer, Wim P Krijnen, Michiel Reneman, Cees P van der Schans

**Affiliations:** 1 Research and Innovation Group in Health Care and Nursing, Hanze University of Applied Sciences, Eysoniusplein 18 9714 CE, Groningen, The Netherlands; 2 Center for Rehabilitation, dept. of Rehabilitation Medicine, University Medical Center Groningen, University of Groningen, Groningen, The Netherlands; 3 Groningen Spine Center, University Medical Center Groningen, University of Groningen, Groningen, The Netherlands; 4 Expertise Center of Health, Social Care and Technology, Saxion Universities of Applied Sciences, Enschede, The Netherlands

**Keywords:** Reference values, Muscle force, Hand-held dynamometer, Maximum voluntary contraction

## Abstract

**Background:**

Muscle force is important for daily life and sports and can be measured with a handheld dynamometer. Reference values are employed to quantify a subject’s muscle force. It is not unambiguous whether reference values can be generalized to other populations. Objectives in this study were; first to confirm the reliability of the utilization of hand-held dynamometers for isometric strength measurement; second to determine reference values for a population of Dutch workers; third to compare these values with those of a USA population.

**Methods:**

462 Healthy working subjects (259 male, 203 female) were included in this study. Their age ranged from 20 to 60 years with a mean (sd) of 41 (11) years. Muscle force values from elbow flexion and extension, knee flexion and extension, and shoulder abduction were measured with the break method using a MicroFet 2 hand-held dynamometer. Reliability was analyzed by calculating ICC’s and limits of agreement. Muscle force expressed in Newton, means, and confidence intervals were determined for males and females in age groups ranging from twenty to sixty years old. Regression equations and explained variances were calculated from weight, height, age, and gender. The mean values and 95% CI were compared to the results from other studies.

**Results:**

Reliability was good; the ICC ranged between 0.83 to 0.94. The explained variance ranged from 0.25 to 0.51. Comparison of data for the Dutch population mean muscle force values with those from the USA revealed important differences between muscle force reference values for the American and Dutch populations.

**Conclusions:**

Muscle force measurements demonstrate a sound reliability. Reference values and regressions equations are made available for the Dutch population. Comparison with other studies indicates that reference values differ between countries.

## Background

Muscle force is considered to be an important determinant for physical performance, activities of daily living, and work or sport performance [1]. Several processes such as aging, development of pathological symptoms, or injury may result in reduced muscle force. Muscle force can be quantified by several viable instruments.

Precise measurements are feasible by employing hand-held dynamometers, which allows muscle force to be measured on a continuous scale. Several authors have demonstrated in various settings that hand-held dynamometry is reliable and the data valid for quantifying muscle force. They ascertained an intraclass correlation coefficient (ICC) of 0.8 or higher, indicating good sound reliability [2-11]. However, precise and reliable measurement outcomes are only meaningful if they can be compared with unaffected muscle groups or, more precisely, with reference values. For example, chronically ill patients may exhibit a bilateral decrease of muscle force. This signifies that the extent of the decline in a specific patient can only be quantified if measured muscle force values are compared with objective reference values. This emphasizes the relevance for the utilization of reference values with which to compare the outcomes of those measurements [8]. However reference values are employed in generally every type of physical examination and are often generated for a specific population. For example, for six minutes walking or pulmonary function tests, reference values are based on a population’s particular origin, and outcomes demonstrate considerable mutual differences [10,11]. Muscle force values, however, are utilized without any ethnic, geographic, or cultural background taken into consideration. Until now, reference values used in clinical practice and in research in the Netherlands are based on populations in the USA. The consideration is justified if reference values for the American population can be generalized to the Dutch population. However, geographical location and cultural backgrounds vary considerably and, therefore, this generalization may not be credible.

The first objective of this study is to confirm the reliability of the use of hand-held dynamometers for isometric strength measurement; the second objective is to determine references values for a population of Dutch workers; the third objective is to compare these values with those of the USA population presented in studies by Bohannon and Andrews [3,9]. Comparison between reference values for muscle force has not been previously performed.

## Methods

### Subjects

The subjects have been employed in several fields, had miscellaneous physical workloads, and were recruited via local press from different localizations in the Netherlands.

Inclusion criteria: Subjects had to meet the inclusion criteria including being between 20 to 60 years of age and working at least 20 hours per week. No absence from work due to illness for more than 2 weeks in the year prior to participation. Subjects were included after providing informed consent and signing a statement of good health after meeting the criteria of the Physical Activity Readiness Questionnaire [12,13]. Exclusion criteria: Subjects were excluded if systolic and diastolic blood pressures exceeded 159 mmHg and 100 mmHg, respectively, as described by the WHO, to prevent cardiovascular injury [14]; were absent from work in the last year as a result of a musculoskeletal disorder; or presented co-morbidities relating to either the cardiovascular or respiratory systems or otherwise did not meet the inclusion criteria. The Medical Ethics Committee of the University Medical Center Groningen, the Netherlands, approved the study protocol.

### Measurement procedure

The subjects’ gender, age, hand dominance, height, weight, physical activity, and Dictionary of Occupational Titles (DOT) level were recorded [15]. The DOT level describes the difficulty of comprehending, nature, tasks of specific types of work, or specified occupational titles. The DOT is meant to match job requirements and employees’ functional abilities and consists of five categories: sedentary, light, medium, heavy, and very heavy.

Maximal isometric voluntary contraction (MVC) was measured with a MicroFet 2 hand-held dynamometer (Hogan Health Industries, Inc. 8020 South 1300 West, West Jordan, USA).

Three consecutive measurements were performed with one minute intervals between contractions. Isometric muscle force from elbow flexion and extension, knee flexion and extension, and shoulder abduction were measured. The protocol consisted of one contraction for every individual muscle in the following sequence; 1 elbow flexion, 2 elbow extension, 3 knee extension, 4 knee flexion, and 5 shoulder abduction. This sequence was performed three times. Observers were allowed to begin left or right according to their preference.

Subjects were asked to gradually increase their muscle force to a maximum effort which would need to be sustained for three seconds. The ‘break technique’ was employed whereby the examiner overpowers the maximum effort of the patient, thereby producing a measurement of eccentric muscle force [16,17].

**Figure 1:**
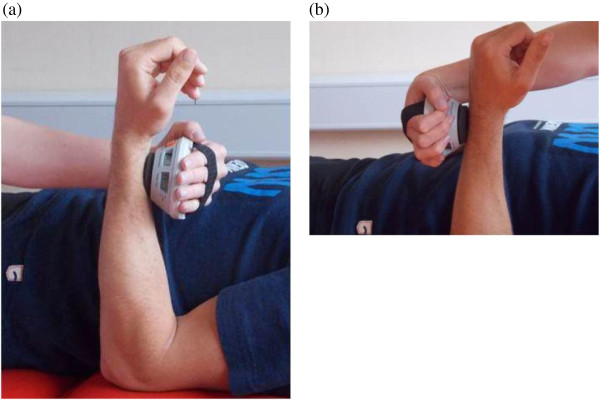
**Positions hand held dynamometer. (a)** elbow flexion, **(b)** elbow extension.

**Figure 2:**
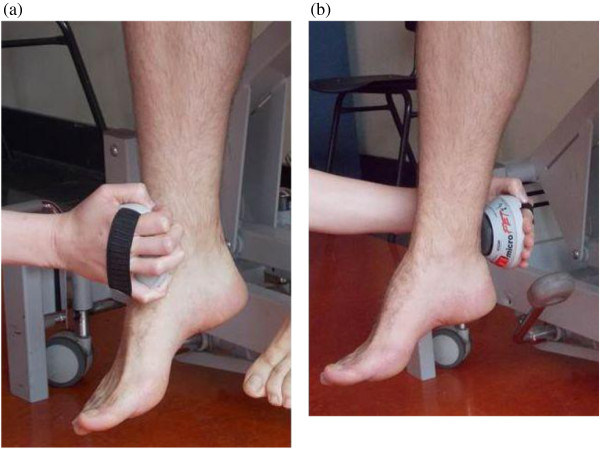
**Positions hand held dynamometer. (a)** Knee extension, **(b)** Knee flexion.

**Figure 3:**
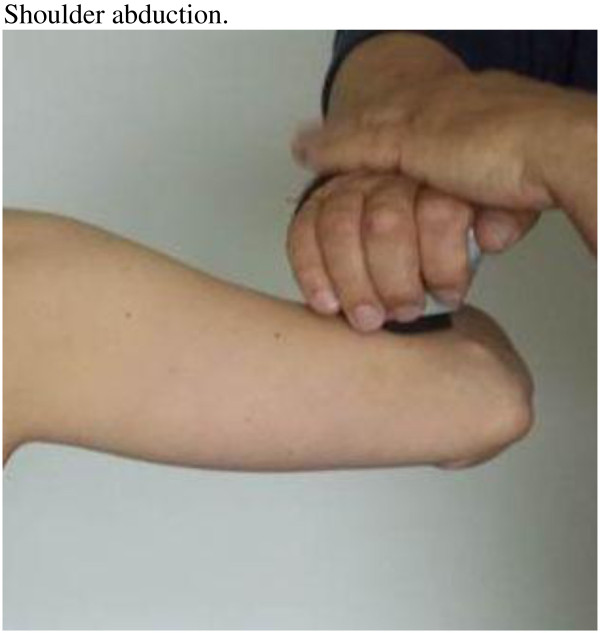
Positions hand held dynamometer shoulder abduction.

The average muscle force of three repetitions was calculated to compensate for and minimize measurement errors. Subjects were assessed by third or fourth year physical therapy students from the Hanze University of Applied Sciences Groningen, the Netherlands. An experienced instructor trained the students prior to the tests. Students were instructed to perform the break technique in the following manner. First, they were instructed to ‘break through’ the subject’s muscle force by countering the force employing a continuous, slow movement. Second, they were to maintain their position and the patient’s position throughout the entire test. Observers provided standardized encouragement. In the event that the observer was unable to break through the patient’s generated force, this was recorded in the administration form, and that result was omitted from the data analysis. Measurements were taken in a standardized and gravity neutral body position. Measurement positions are described in Table 1 (Figures 1, 2 and 3).

**Table 1:** Description of body positions during measurements

**Muscle force/movement**	**Joint/Limb position**	**Localization HHD**	**Position subject**	**Fixation subject**	**Position/fixation observer**
**Elbow flexion**	Neutral shoulder, elbow flexed 90°; upper arm against trunk	Just proximal to styloid process of radius	Lying supine	By body weight; feet against wall	Alongside the table and test subject, leaning backward
**Elbow extension**	Same as in flexion	Just proximal to ulnar head	Same as in flexion	Same as in flexion	Same as in flexion
**Knee flexion**	Hip and knee flexed 90°	Just proximal to calcaneus	Sitting on table	By body weight and active fixation while gripping table	In front of test subject; feet fixed onto table
**Knee extension**	Same as in flexion	Just proximal to talis	Same as in flexion	By body weight and active fixation.	In front of test subject; fixated by body weight, gripping table, and pushing forward; HHD fixation against upper leg
**Shoulder abduction**	90° abduction in shoulder	Just proximal to lateral epicondyle	Sitting on examination table or chair	Body weight	Behind subject

HHD, Hand-held dynamometer.

### Statistical analyses

All data were analyzed with SPSS 14.0. To answer the primary objective of this study, reliability of the three repeated measurements, the intraclass correlation coefficients (ICC) two-way random effects model including lower and upper confidence limits (LCL and UCL), as well as the limits of agreement (LOA) were calculated [18]. Limits of agreement were collectively calculated for males and females and encompassing all age groups and for each pair of repeated measurements [19]. ICCs were interpreted as follows: ICC < 0.25 is low reliability; 0.25 < ICC < 50 moderate reliability; 50 < ICC < 75 good reliability and ICC > 0.75 is excellent reliability [19-21].

To address the second objective, reference values for muscle force were constructed by calculating means and standard deviations. Results are stratified by age groups and gender. Differences between males and females were analyzed utilizing independent t-tests. To investigate the degree to which muscle force is linearly related to four independent variables, i.e., gender, weight, height and age, a linear regression analysis was performed. Due to these regression equations and the explained variance, the r^2^ was calculated. To answer the third objective, comparisons between muscle force outcomes of the current study and two different studies were performed by comparing the means of the two other studies with our means and a 95% confidence interval (95%CI) [3,9].

## Results

### Subjects

A sample of 462 healthy subjects (259 males and 203 females) was included in this study.

The subject and group characteristics are presented in Table 2.

**Table 2:** Characteristics of the population stratified by age group and gender

**Male**	20-59	20-29	30-39	40-49	50-59
Age	41.7(11)	25.2(3)	33.6(3)	44.9(3)	54.1(3)
Height	182.1(8)	182.0(8)	181.4.6(8)	183.5(8)	181.2(7)
Weight	80.8(12)	74.3(10)	80.6(13)	82.4(10)	82.4(14)
BMI	24.4(4)	22.4(3)	24.4(4)	24.4(3)	25.1(4)
DOT 1	18.9	10.0	22.0	25.4	15.4
DOT 2	44.3	33.3	24.3	46.0	60.0
DOT 3	22.9	43.3	34.1	12.7	16.9
DOT 4	13.9	13.3	19.5	15.9	7.7
**Female**	20-59	20-29	30-39	40-49	50-59
Age	40.2(10)	25.9(3)	34.8(3)	44.3(3)	53.6(3)
Height	170.1(7)	172.5(6)	170.8(8)	170.0(7)	167.4(7)
Weight	68.0(11)	68.1(13)	68.2(9)	68.0(11)	67.8(12)
BMI	23.4(3)	22.9(4)	23.5(3)	23.4(3)	24.1(4)
DOT 1	21.2	14.0	21.1	12.7	43.6
DOT 2	42.5 41.9	33.4	54.0	33.3	
DOT 3	35.3	44.2	45.5	30.2	23.1
DOT 4	1	0	0	3.1	0

Age, Height, Weight and BMI expressed in mean(sd) and DOT is expressed in percentage of the population.

DOT; Dictionary of Occupational Titles. DOT 1 = sedentary, DOT 2 = light, DOT 3 = medium, DOT 4 = heavy/very heavy.

### Reliability

Correlations between the different measurements vary between 0.83 and 0.94, ICC values vary between 0.83 and 0.92 and are presented in Table 3. Since the confidence intervals were small, it is relatively certain that the population values of the coefficients are similar to the estimated values. All ICC values were higher than 0.75, indicating good reproducibility for all ten muscle measurements [19,21]. The limits of agreement varied between 37.0 and 117.8 Newton. Elbow extension left demonstrates a small 95%CI while knee extension right exhibits a large range of the 95%CI.

**Table 3:** Correlation between the three measurements, intraclass correlation coefficient, limits of agreement, for three repeated measurements

**Muscle force**	**Corr1-2**	**Corr1-3**	**Corr2-3**	**ICC**	**LCL- UCL**	**LOA1-2**	**LOA1-3**	**LOA2-3**
Elbow flex. left	0.88	0.87	0.87	0.87	0.85 - 0.89	± 59.4	± 61.6	±61.0
Elbow flex. right	0.85	0.86	0.89	0.87	0.85 - 0.89	± 67.8	± 66.8	± 56.3
Elbow ext. left	0.89	0.85	0.88	0.88	0.86 - 0.89	± 42.8	± 50.3	±44.1
Elbow ext. right	0.91	0.91	0.93	0.92	0.90 - 0.93	± 37.0	± 37.2	±32.9
Knee flex. left	0.83	0.81	0.87	0.84	0.81 - 0.86	± 75.9	± 82.5	±68.8
Knee flex. right	0.87	0.82	0.88	0.86	0.83 - 0.88	± 69.8	± 81.4	±67.8
Knee ext. left	0.88	0.85	0.91	0.88	0.86 - 0.89	± 104.0	±117.8	±94.3
Knee ext. right.	0.91	0.90	0.94	0.92	0.90 - 0.93	± 96.3	± 106.7	± 80.2
Abduction left	0.83	0.80	0.87	0.83	0.80 - 0.86	± 55.2	± 59.5	±48.4
Abduction right	0.85	0.87	0.92	0.88	0.85 - 0.90	± 52.6	± 46.5	±37.3

Corr: Correlation, ICC: intraclass correlation coeficient, LCL: lower control limit, UCL: upper control limit, LOA: limits of agreement.

### Reference values

Tables 4 and 5 illustrate the mean muscle force values for reference values from elbow flexion and extension, knee flexion and extension, and shoulder abduction stratified by age groups, gender, and dominance. Regression equations and explained variance are presented in Table 6. Regression equations were calculated with height, weight, age, and gender. The explained variance varies between 0.25 for knee extension right and 0.51 for elbow extension left.

**Table 4:** Dominant and non-dominant muscle strength means (sd) per age group for males

**Male**		**Dominant**		**Non dominant**	
**Muscle force**	**Age group**	**N**	**Mean(Sd.)**	**N**	**Mean (Sd.)**
Elbow flexion	20-29	48	281(48)	48	261(49)
	30-39	51	273(50)	51	266(51)
	40-49	70	271(59)	70	261(51)
	50-59	59	259(52)	59	245(47)
Elbow extension	20-29	48	186(38)	48	182(37)
	30-39	51	183(40)	51	179(45)
	40-49	70	185(46)	70	179(44)
	50-59	59	181(37)	59	173(36)
Knee flexion	20-29	48	267(57)	48	252(52)
	30-39	51	262(60)	51	250(55)
	40-49	68	274(77)	69	263(77)
	50-59	59	242(57)	59	234(55)
Knee extension	20-29	47	379(105)	47	371(112)
	30-39	51	351(99)	51	341(101)
	40-49	69	368(114)	70	341(107)
	50-59	59	337(103)	57	335(102)
Schoulder abduction	20-29	14	172(48)	14	173(35)
	30-39	26	181(38)	26	176(40)
	40-49	35	173(43)	35	177(40)
	50-59	37	178(39)	39	177(43)

**Table 5:** Dominant and non-dominant muscle strength means (sd) per age group for females

**Female**		**Dominant**		**Non dominant**	
**Muscle force**	**Age group**	**N**	**Mean(Sd.)**	**N**	**Mean(Sd.)**
Elbow flexion	20-29	51	191(30)	51	183(30)
	30-39	39	195(34)	39	186(35)
	40-49	66	191(37)	66	186(37)
	50-59	34	181(29)	34	166(22)
Elbow extension	20-29	51	132(28)	51	131(28)
	30-39	39	128(24)	39	125(26)
	40-49	66	131(28)	66	125(29)
	50-59	34	120(20)	34	118(27)
Knee flexion	20-29	51	198(38)	51	191(37)
	30-39	39	190(41)	39	188(35)
	40-49	66	190(51)	67	183(52)
	50-59	34	174(42)	34	169(45)
Knee extension	20-29	51	261(80)	51	260(75)
	30-39	38	273(87)	39	264(88)
	40-49	66	262(127)	67	245(79)
	50-59	34	244(66)	34	228(51)
Schoulder abduction	20-29	14	115(19)	14	124(23)
	30-39	22	116(26)	22	118(30)
	40-49	41	119(28)	41	118(26)
	50-59	15	114(22)	15	116(21)

**Table 6:** Regression equations for calculation of reference values

**Muscle force**	**Regression equations**	**R**^**2**^
Elbow flexion left	-4.93 + 56.96*S-0.64*A + 0.89*W + 0.89*H	0.51
Elbow flexion right	10.67 + 57.47*S-0.72*A + 0.95*W + 0.85*H	0.49
Elbow extension left	23.85 + 36.56*S-0.50*A + 1.07*W + 0.29*H	0.44
Elbow extension right	80.39 + 41.56*S-0.47*A + 1.14*W-0.06*H	0.48
Knee flexion left	47.92 + 43.52*S-0.60*A + 1.36*W + 0.40*H	0.34
Knee flexion right	43.84 + 47.03*S-0.71*A + 1.33*W + 0.50*H	0.35
Knee extension left	-204.36 + 43.69*S-1.13*A + 1.90*W + 2.19*H	0.31
Knee extension right	-215.54 + 40.73*S-0.82*A + 2.0*W + 2.22*H	0.25
Shoulder abduction left	-20.68 + 45.25*S-0.04*A + 0.64*W + 0.56*H	0.46
Shoulder abduction right	10.07 + 43.63-0.16*A + 0.76*W + 0.36*H	0.43

A, age; W, weight; H, height; S, sex (1 for male, 0 for female).

### Comparison

Mean muscle force values and the 95% CI from the current study and mean muscle force values from studies by Bohannon and Andrews are presented in Tables 7 and 8. Comparison of Dutch mean muscle force values to those from Bohannon and Andrews [3,9] revealed that an significant difference exists between reference muscle force values between different populations.

**Table 7:** **Comparison between the present study and studies of Bohannon**[3]**and Andrews**[9]**for male**

**Male**	**Dominant**			**Non dominant**		
**Muscle force**	**Douma**	**Bohannon**	**Andrews**	**Douma**	**Bohannon**	**Andrews**
	**Mean (95% CI)**	**Mean**	**Mean**	**Mean (95% CI)**	**Mean**	**Mean**
**Elbow flexion**						
**Age group**						
20-29	281 (267-295)	285	-	261 (247-276)	**279**	-
30-39	273 (259-287)	269	-	266 (252-281)	281	-
40-49	271 (258-286)	269	-	261 (249-274)	270	-
50-59	259 (246-272)	**287**	**292**	245 (232-257)	**268**	**272**
**Elbow extension**						
**Age group**						
20-29	186 (175-197)	**244**	-	182 (171-194)	**245**	**-**
30-39	185 (172-194)	**214**	-	179 (167-192)	**231**	**-**
40-49	185 (174-196)	**210**	-	179 (169-190)	**214**	-
50-59	181 (171-190)	**19**7	188	173 (164-182)	**186**	178
**Knee extension**						
**Age group**						
20-29	379 (348-409)	**575**	-	371 (339-404)	**579**	-
30-39	351 (323-378)	573	-	341 (312-369)	**572**	-
40-49	368 (341-395)	**583**	-	341 (315-366)	**589**	-
50-59	337 (310-363)	**471**	448	335 (308-362)	**468**	**-439**
**Shoulder abduction**						
**Age group**						
20-29	172 (144-200)	**258**	-	173 (152-193)	**246**	-
30-39	181 (165-196)	**249**	-	176 (159-192)	**237**	-
40-49	173 (158-188)	**246**	-	177 (163-191)	**244**	-
50-59	178 (165-191)	**240**	238	177 (163-191)	**223**	**-222**

Bold printed numbers are values outside the 95%CI of this study.

Age groups are given in decades in years, Muscle force is given in Newtons, LCL = lower control limit, UCL upper control limit, – not available.

**Table 8:** **Comparison between the present study and studies of Bohannon**[3]**and Andrews**[9]**for female**

**Female**	**Dominant**			**Non dominant**		
**Muscle force**	**Douma**	**Bohannon**	**Andrews**	**Douma**	**Bohannon**	**Andrews**
	**Mean (95%C)**	**Mean**	**Mean**	**Mean (95%C)**	**Mean**	**Mean**
**Elbow flexion**						
**Age group**						
20-29	191 (182-199)	**155**	-	183 (175-192)	**151**	-
30-39	195 (184-206)	**164**	-	186 (175-198)	**161**	-
40-49	191 (182-199)	**151**	-	186 (176-195)	**157**	-
50-59	181 (171-191)	**155**	167	166 (158-174)	**156**	160
**Elbow extension**						
**Age group**						
20-29	132 (124-139)	**116**	-	131 (123-139)	**115**	-
30-39	128 (121-135)	**117**	-	125 (116-133)	**119**	-
40-49	131 (124-137)	**110**	-	125 (118-132)	**112**	-
50-59	120 (113-127)	**111**	108	118 (109-127)	**107**	104
**Knee extension**						
**Age group**						
20-29	261 (234-288)	**467**	-	260 (238-281)	**466**	-
30-39	273 (244-302)	**408**	-	264 (235-292)	**411**	-
40-49	262 (231-293)	**381**	-	245 (225-265)	**363**	-
50-59	244 (221-267)	**335**	298	230 (210-246)	**319**	293
**Shoulder abduction**						
**Age group**						
20-29	115 (104-127)	**153**	-	124 (110-137)	**135**	-
30-39	116 (105-128)	**139**	-	118 (104-131)	**136**	-
40-49	119 (110-128)	**139**	-	118 (109-126)	**129**	-
50-59	114 (110-128)	**137**	135	116 (104-128)	**135**	124

Bold printed numbers are values outside the 95%CI of this study.

Age groups are given in decades in years, Muscle force is given in Newtons, LCL = lower control limit, UCL upper control limit, – not available.

Comparison indicates that, for males, mean muscle force values of Bohannon and Andrews are greater than those of the current study except for elbow flexion of the dominant and non-dominant sides in which only the age group 50–59 years exhibits greater values.

For females, mean muscle force values of Bohannon and Andrews were lower for elbow flexion and extension than those in the present study with the exception of elbow extension, non-dominant for age group 30 to 39 (Bohannon) and 50 to 59 (Andrews) years.

Shoulder abduction and knee flexion and extension indicated greater values in the study of Bohannon and Andrews, except for shoulder abduction of the non-dominant side with age group 20 to 29 years.

## Discussion

Reliability of muscle force measurements with a hand held dynamometer is good to excellent. All ICC values exceeded the criterion of 0.80, indicating good reliability for all ten muscle measurements. These findings corroborate with those of Bohannon [3]. The LOA, however, varies substantially.

Reference values for muscle force for the Dutch working population between 20 and 60 years of age are now made available. Reference values including age gender, weight and height can be calculated with regression analysis as independent predictors for muscle force.

Comparison of the Dutch mean muscle force values to those published by Bohannon and Andrews revealed significant differences between reference values for muscle force values between the assessed populations. Comparison of reference values between populations have not been initiated previously.

Muscle force measurements with a hand held dynamometer exhibit a good reliability as demonstrated by the ICC. The LOA, however, vary substantially. Muscle groups with a relatively low muscle force demonstrate a small range of the LOA while muscle with a greater muscle force exhibit a larger range of the LOA, indicating that measurements of stronger muscles are less precise. Though hand held dynamometers have shown to be a reliable and beneficial instrument for measuring muscle force, a hand held dynamometer may possess some practical limitations. In subjects with high Quadriceps muscle force, it might be impossible to perform a correct measurement. During our study, it was not possible to perform a correct measurement of the Quadriceps muscle in six subjects due to high muscle force as observers were not capable of performing a correct break procedure. As reliability and validity may be affected during these measurements, bias was likely present, which is the reason that these results were omitted from the analysis. The influence of exclusion of these data on reliability, regression formulas, and reference values is very limited due to the considerable sample size. Provided that observers were able to properly perform according to the protocol, the regressions formula for knee extension might be only slightly changed.

In our opinion, a hand-held dynamometer is not suitable for measuring Quadriceps muscle force in stronger subjects.

Reference values for muscle force for the Dutch working population between the ages of 20 and 60 years are now made available. Regression equations illustrate that gender, weight, and height are of major influence on muscle force. The effect of age, however, is limited. In several of the regression analyses, the effect of age was small, though significant, due to the considerable sample size. Regression analysis demonstrated that the effect of aging for subjects aged 20–60 years is larger for lower extremities than for upper extremities. These results are predominantly consistent with previously reported results [3,9]. Bohannon and Andrews also reported that gender, age, height, and weight are predictors of muscle force and that age correlated significantly, though very limited, with muscle force. Comparison of the outcomes of our study to those earlier exhibited an important difference between reference values. The differences in upper extremity tests, however, were moderate in all cases, whereas most of the lower extremity differences were considerable. For instance, differences in muscle force greater than 100 Newton for knee extension may have clinical consequences as 100 Newton’s may be up to 43 percent of the maximum knee extension force in the Dutch female population. The observed differences, however, exceed 100 Newton. This is all the more remarkable because, in our study, we employed the break method while, in the studies of Bohannon and Andrews, the make method is used. The break method may lead to higher levels in muscle strength measurements [16]. The observed differences in the lower extremity are relevant for clinical practice. It appears to be evident that these differences may probably cause unattainable and/or undesirable training goals to be set and may result in undesired side effects as these external reference values may be too high and, therefore, not suitable for the Dutch population. However, reference values formulated for the United States are, at this moment, utilized in clinical practice and research in the Netherlands.

The results of our study demonstrate that reference values cannot simply be generalized to any country, geographical area, or population. Therefore, it is necessary to generate reference values for different countries or geographical areas. For other physiological tests such as the six minutes walking test reference values for specific geographic reference values are available and indicate considerable differences [10]. Although we did not assess cultural habits or demographic aspects of populations, it is likely that the outcomes of muscle force measurements may be influenced by several such factors. Psychological state or prior experiences related to exertion or physiological responses to exercise, exertion, or pain might have influenced the outcomes [22]. In addition, body composition and weight are related to muscle force as presented in the regression equations. Another potential explanation for the differences between our reference values and those previously reported by Andrews and Bohannon is the difference in time periods. The reference values of Andrews and Bohannon were determined in 1996 and ours in 2010. In approximately 20 years, some characteristics such as BMI may have changed which may affect references values equations.

### Study limitations

In our study, we only tested the employed working population between the ages of 20 and 60 years. Our study, therefore, only provides reference values and comparison for this group. Our study does not provide information regarding, for example, unemployed businessmen or housekeepers. Another limitation in our study is that observers were male and female. We did not register whether subjects were tested by male or female observers. The outcomes of measurements may be biased by the gender of the observer.

Reliable muscle force measurements, appropriate and applicable reference values, and accurate knowledge of acquired muscle force in daily living facilitates formulating an effective and accurate rehabilitation process with clear and realistic goals and objective effects.

Although reliable measurements of a person’s muscle force are beneficial, no reliable procedures are currently available for translating isometric contractions or reference values, for that matter, into function. Functional tests probably provide an improved reflection of a subject’s functional muscle force, capacity, or ability for activities of daily living or work. This probably indicates that the role of muscle force should be interpreted with caution and that other variables may also influence activities of daily living. Additional studies are needed to define the specific role and the amount of muscle force required in activities of daily living.

## Conclusions

Measuring muscle force by dynamometry is reliable and suitable for clinical practice. Substantial differences exist for reference muscle force values between different populations. Reference values are specific for different regions and cannot simply be generalized to other populations.

## Abbreviations

MRC: Medical research council; MVC: Maximal voluntary contraction.

## Competing interests

The authors declare that they have no competing interests.

## Authors’ contributions

R.S Performed the actual measurements and instructed other observers. Participated in in the design and coordination of the study. Made several intellectual contributions to the draft of the manuscript. W.K. Conceived and performed and coordinated the majority of the statistical analyses. Made several intellectual contributions to the design and draft of the manuscript. M.R. Made several intellectual contributions to the draft of the manuscript and participated in in the design and coordination of the study. C.S. Made several intellectual contributions to the design and draft of the manuscript. Participated in the statistical analyses. All authors read and approved the final manuscript.

## Pre-publication history

The pre-publication history for this paper can be accessed here:

http://www.biomedcentral.com/2052-1847/6/10/prepub
